# Supplementary material for: Structural characterisation of the Chaetomium thermophilum Chl1 helicase
Source: PLoS One. 2021 May 10;16(5):e0251261. doi: 10.1371/journal.pone.0251261 (PMC8109800; doi:10.1371/journal.pone.0251261)
Supplement: S1 Raw Images — (PDF) [file pone.0251261.s001.pdf]

S1 Raw Images

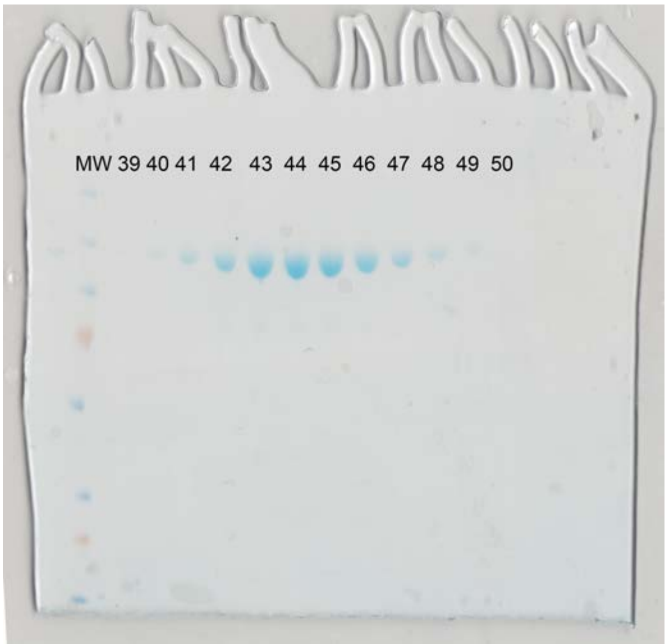

Scan of orignal gel.
Gel fitration fractions of *C. thermophilum* Chl1.
Relates to Figure 1B.

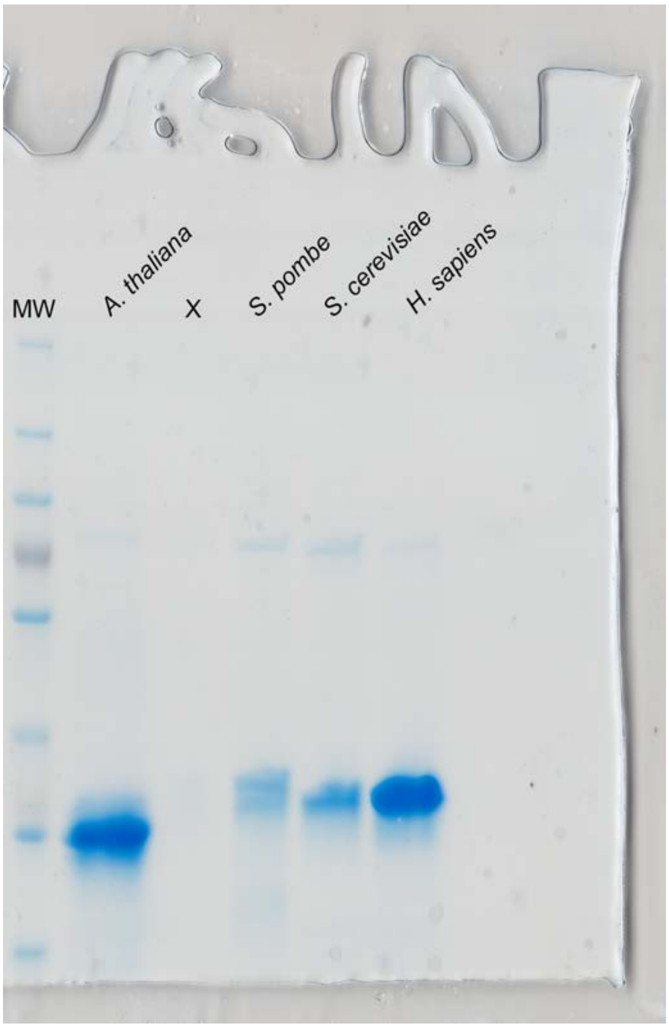

Scan of orignal gel.
Expression of inserts.
Relates to Figure 4C.

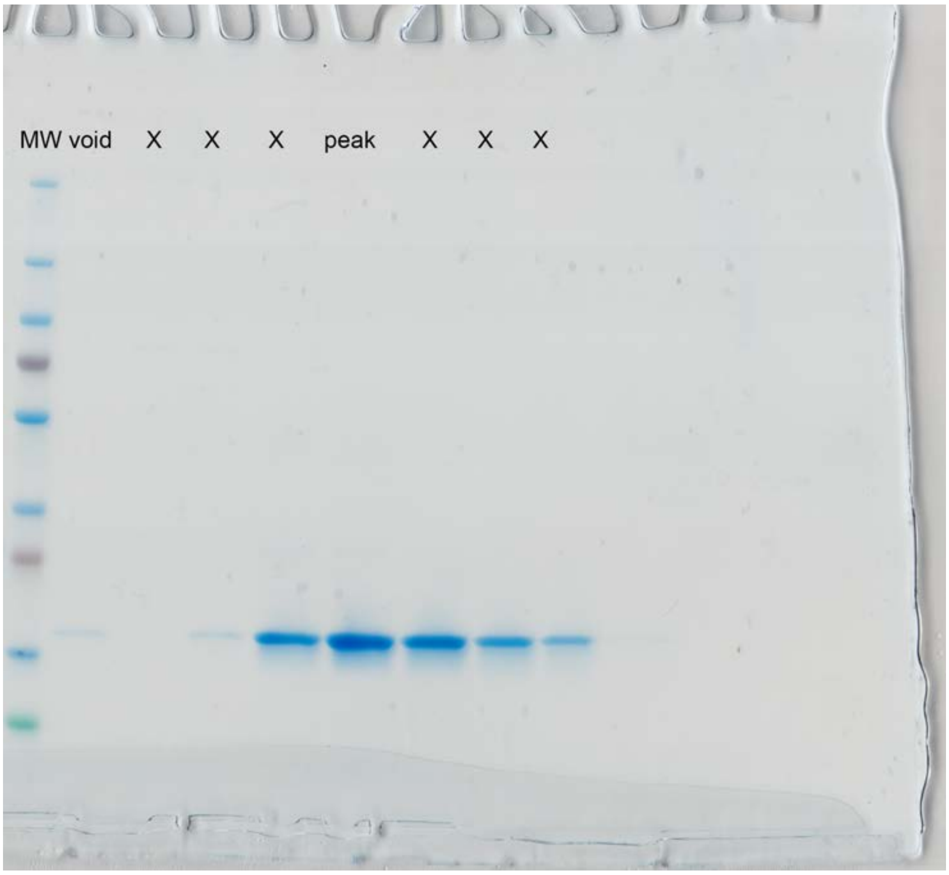

Scan of orignal gel.
Gel fitration fractions of *C. thermophilum* Chl1 insert.
Relates to Figures 4C and 4D.

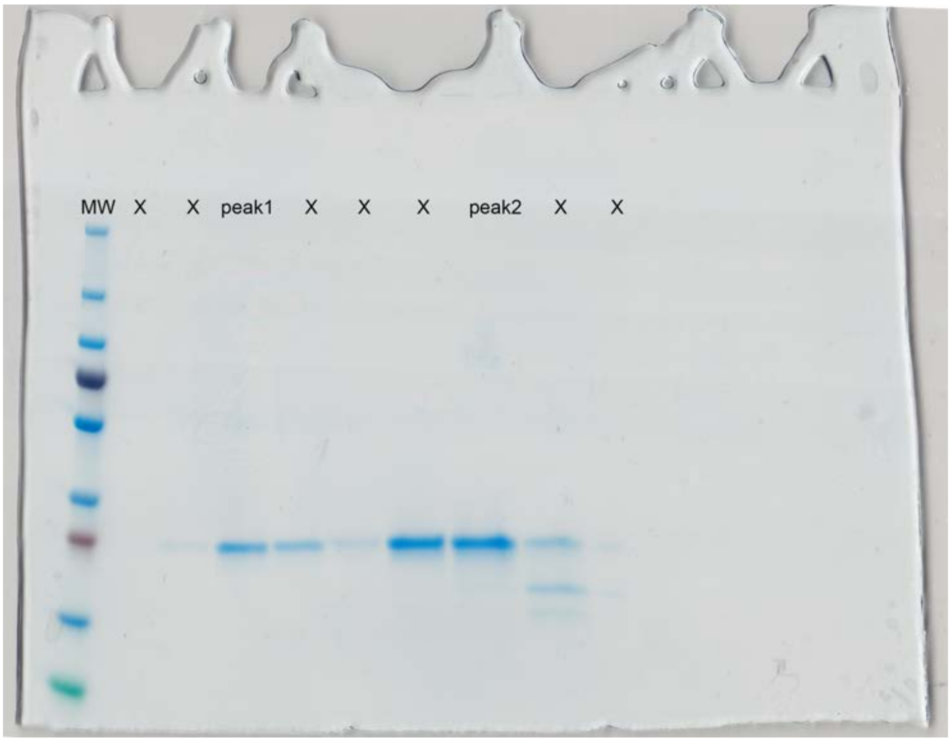

Scan of orignal gel.
Gel fitration fractions of *H. spapiens* Chl1 insert.
Relates to Figure 4D.
