# Supplementary material for: Structural characterisation of the Chaetomium thermophilum Chl1 helicase
Source: PLoS One. 2021 May 10;16(5):e0251261. doi: 10.1371/journal.pone.0251261 (PMC8109800; doi:10.1371/journal.pone.0251261)

Structural characterisation of the *Chaetomium thermophilum* Chl1 helicase

Zuzana Hodáková, Andrea Nans, Simone Kunzelmann, Shahid Mehmood, Ian Taylor, Frank Uhlmann, Peter Cherepanov, Martin R. Singleton

SUPPLEMENTARY INFORMATION

Supplementary Table S1

Supplementary Figure Legends

Supplementary Figures S1-S3

Construct abbreviation	Protein name	Gene species	Sequence
Chl1	Chl1	*C. thermophilum*	$Chl1^{FL}$
$MiniChl1^{V1}$	Chl1	*C. thermophilum*	$Chl1^{1-74 +216-918}$
$MiniChl1^{V2}$	Chl1	*C. thermophilum*	$Chl1^{1-143 + 208-918}$
*Ct*Chl1 Insert	Chl1	*C. thermophilum*	$Chl1^{75-213}$
*At*Chl1 Insert	Chl1	*A. thaliana*	$Chl1^{51-188}$
*Hs*Chl1 Insert	Chl1	*H. sapiens*	$Chl1^{56-226}$
*Sp*Chl1 Insert	Chl1	*S. pombe*	$Chl1^{56-217}$
*Sc*Chl1 Insert	Chl1	*S. cerevisiae*	$Chl1^{54-227}$

Supplementary Table S1. List of constructs used in this study.

SUPPLEMENTARY FIGURE LEGENDS

Supplementary Figure S1. Data processing pipeline for the 7.7Å reconstruction.

Supplementary Figure S2. Final model resolution. (**A)** The local resolution map of the structure, with the highest resolution of 7Å obtained. (**B)** 3D Fourier Shell Correlation (3DFSC) for of the map.

Supplementary figure S3. PsiPred predictions for insert regions of Chl1. Proteins from three different species are shown. Ct – *Chaetomium thermophilum*, Sc – *Saccharomyces cerevisiae*, Hs – *Homo sapiens*. Pink bars represent α helices, yellow bars represent β strands, grey bars represent coils. The first and last amino acid of the insert in each species is underlined with a blue bar.

Hodakova *et al.*, Supplementary Figure S1.

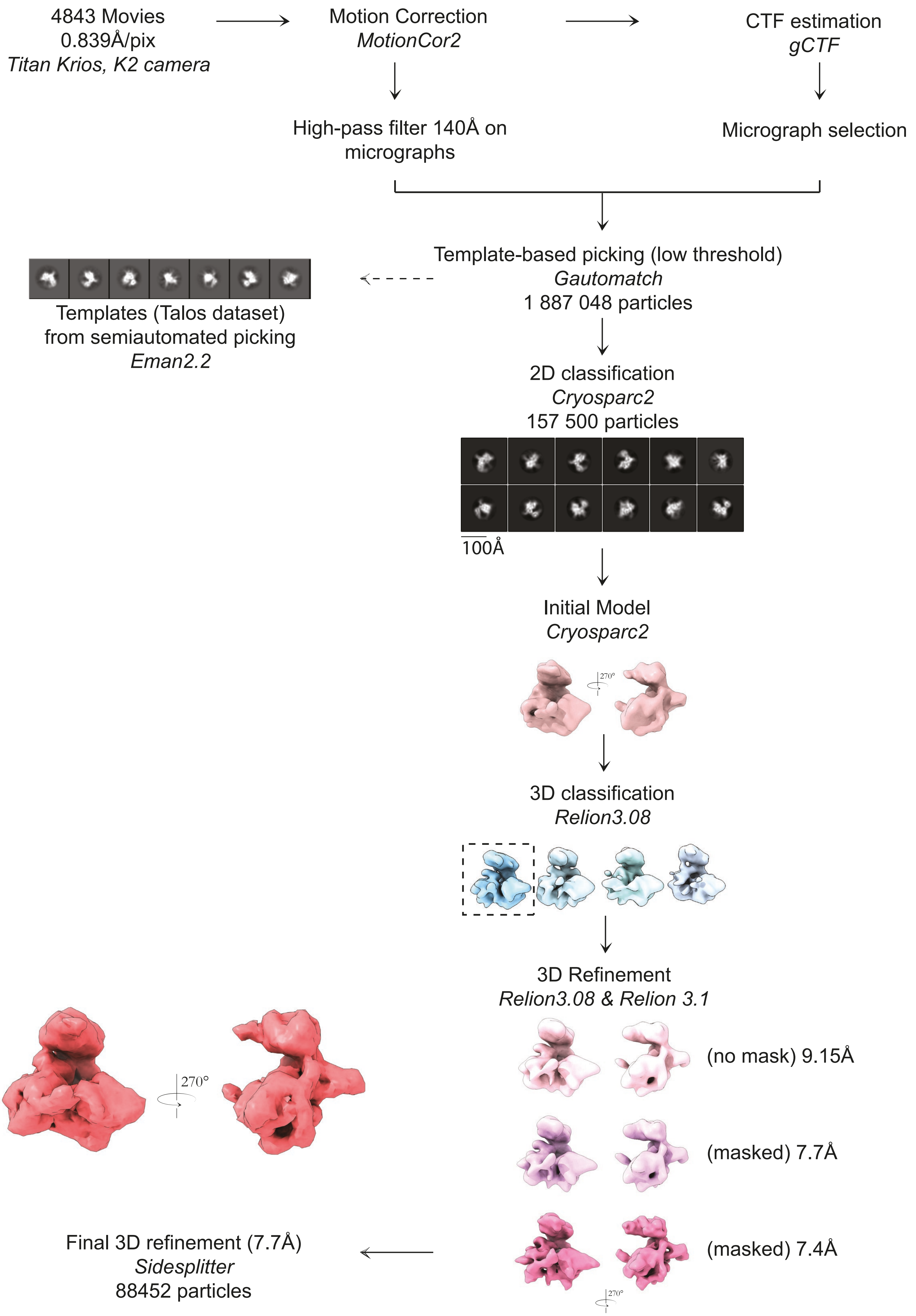

Hodakova *et al*., Supplementary Figure S2.

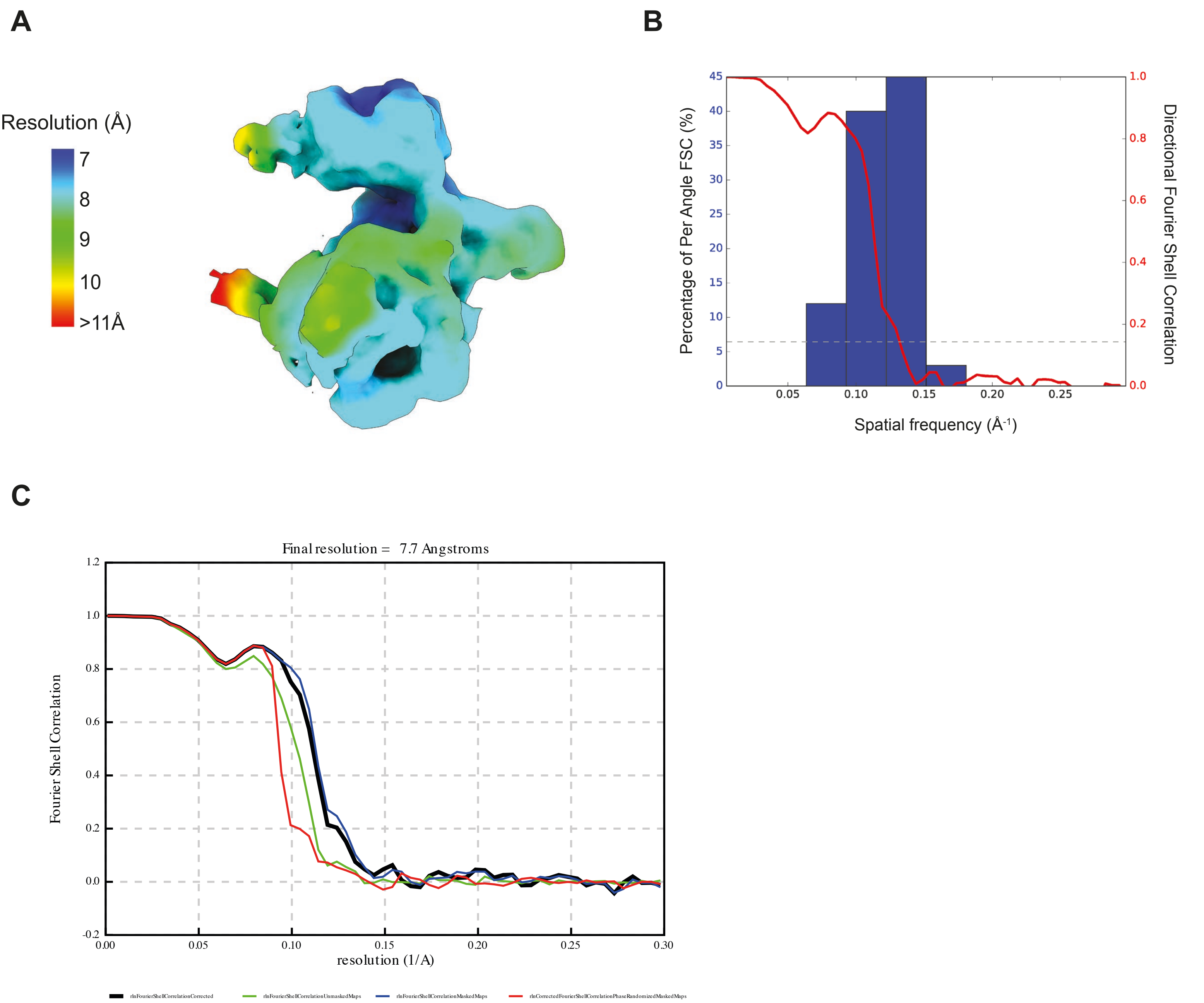

Hodakova *et al.*, Supplementary Figure S3.

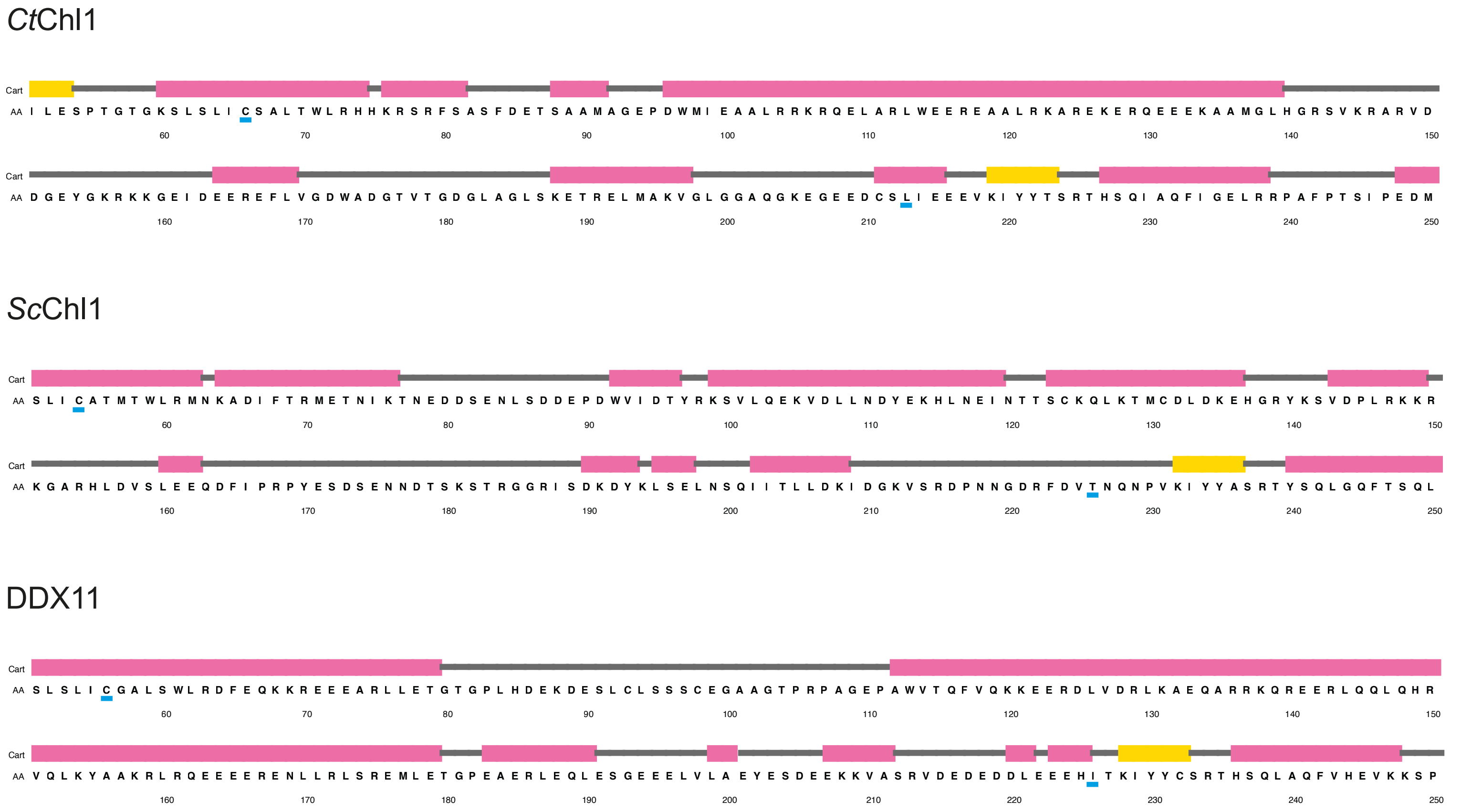

Supplement: S1 File — (PDF) [file pone.0251261.s002.pdf]
