# Supplementary material for: Structural characterisation of the Chaetomium thermophilum Chl1 helicase
Source: PLoS One. 2021 May 10;16(5):e0251261. doi: 10.1371/journal.pone.0251261 (PMC8109800; doi:10.1371/journal.pone.0251261)
Supplement: Attachment — Submitted filename: responses.docx [file pone.0251261.s003.docx]

**Responses to Referees**

(line numbers refer to revised, non-tracked manuscript)

**General comments from AE:**

The rationale behind using the fungus Chaeotmium thermophilum Chl1 gene (instead of the S cerevisiae gene discussed in the introduction) needs to be explicitly described.  It’s relationship to the yeast gene needs to be discussed in terms of identity and function. In addition, for clarity, please include the organism in the title to the paper.

We have expanded on the rationale for using this Chl1 ortholog, and further explained its relationship to the yeast gene (lines 108-115). We have amended the title as requested.

Are there any means for providing better reproductions of the rather blurry E-M data shown in Figure2?

The blurriness of the EM images shown (especially in Fig. 2B) reflect the fundamentally limited resolution of the experimental data, so there is unfortunately no way we can improve on these.

Please place the cartoon describing the background regarding the Fe-S closer currently in the Supplementary Material into the main set of Figures as indicated by the Reviewer.

We have incorporated the cartoon illustration of the Chl1 domains including the Fe-S cluster into Figure 1.

Please note the inaccurate order of the panels in Figure 4.

This has been corrected in the figure legend.

Introduction Line 57: Define CMG;  Line 66: in yeast: Has the process been elucidated in other organisms?

We have defined CMG in the text (line 57). The CMG is a conserved amongst eukaroytes, but the extent of functional conservation of Chl1 is unclear (lines 66-68).

Results;' Line 120: Despite extensive efforts, (add comma); Line 141: Change “predictions suggesting” to “predictions and suggesting”; Line 147: Change “to nucleotide-free” to “to the nucleotide-free”; Line 181-182: Explain meaning of “the C. thermophilum ? and DDX11 sequences”.  Are you comparing organisms or genes?

All these have been corrected and the section on the insert sequences re-worded (now lines 189-190).

**Reviewer #1**

Text errors:

Line 30: “alterarations”
Line 31: “a the”
Line 135: “suugests”
Line 136: ‘minimsing’

All text errors have been corrected.

**Reviewer #2**

Last sentence of Abstract: “suggesting a the insert domain…” Something grammatically wrong here with inclusion of both “a” and “the”

Corrected.

Line 64: “role of Chl1/DDX1 in SCC…” It appears that DDX1 should be DDX11
The abstract should state that it is a 7.7 Angstrom cryo-EM structure which has been solved.

Both corrected.

Line 251: The authors state that full-length Chl1 has very low affinity for DNA. Does this correspond to data not shown? Can these data be included in the Supp Data section?

This corresponds to the low affinity observed in our FP binding assay. The wording has been slightly altered to clarify this (line 260).

Line 59-60: Previously published work demonstrated that Timeless stimulates DDX11 helicase activity. Relevant reference: Cali et al., Nucleic Acids Research (2016) 44(2):705-17.

Reference included.

Lines 82-84: “20 kDa domain inserted in helicase domain I (HDI).” Because authors make a number of comparison statements of Chl1 and the domains such as the insert in HD1 to other Fe-S helicases including XPD and FANCJ, it is advised that a cartoon alignment of Fe-S cluster helicases being discussed in this work (e.g.., DDX11, FANCJ, RTEL1, XPD) along with Chl1 be included as a figure that is present in the manuscript (not Supp Data). The inclusion of this figure (as a sole figure or a panel of a figure) will help to orient the reader and extrapolate information/insights from the current study to other Fe-S cluster helicases being discussed in the manuscript.

We have included a cartoon schematic of the XPD family sequences and approximate structural arrangement in figure 1A, highlighting the domain organisation and Fe-S location.

**Reviewer #3:**

1) In the introduction and abstract, multiple acronyms are defined that are never used again (ex. “FPC”, “CIP”, “G4”, etc). These should be taken out unless they are used later in the text.

This has been done. “FPC” is used later in the revised text.

2) In line 72, there is one spelled out number (“one”) and one digit number (“3”). The authors should be consistent with this.

Corrected.

3) The panels in figure 4 are out of order and referenced incorrectly in the text and multiple figure legends.

Corrected.

4) In Figure 4C and related text it would be helpful to include the sequence numbers of the insert.

The sequence numbers of the insert are now shown in the figure and the legend.

5) Also, in Figure 4C, it is unclear what the gray bars are referring to. If they are not being included in the graph, why are they shown?

The gray bars show peptides that were detected in the MS experiments, but not shown on the graph because they represented areas of multiple coverage (for the sake of clarity, only single peptides are shown in the graph). As they add no useful data to the figure, we have removed them entirely.

6) In line 234, “regions” is misspelled.

Corrected.

7) The sentence that begins with “In higher eukaryotes…” in line 540 should be in the main text, not in the figure legend.

We have removed the sentence from the figure legend. The details of the inhibition by MAT1 are included in the discussion (lines 266-270).

8) The sentence that begins with “It is possible that the human…” in line 555 should also be in the main text and not in the figure legend.

Corrected.
